# Chaos of *Wolbachia* Sequences Inside the Compact Fig Syconia of *Ficus benjamina* (*Ficus*: Moraceae)

**DOI:** 10.1371/journal.pone.0048882

**Published:** 2012-11-08

**Authors:** Chun-Yan Yang, Jin-Hua Xiao, Li-Ming Niu, Guang-Chang Ma, James M. Cook, Sheng-Nan Bian, Yue-Guan Fu, Da-Wei Huang

**Affiliations:** 1 College of Plant Protection, Shandong Agricultural University, Tai’an, China; 2 Key Laboratory of Zoological Systematics and Evolution, Institute of Zoology, Chinese Academy of Sciences, Beijing, China; 3 Environment and Plant Protection Institute, Chinese Academy of Tropical Agricultural Sciences, Danzhou, Hainan, China; 4 School of Biological Sciences, University of Reading, Reading, Berkshire, United Kingdom; 5 Hawkesbury Institute for the Environment, University of Western Sydney, Sydney, Australia; University of Leeds, United Kingdom

## Abstract

Figs and fig wasps form a peculiar closed community in which the *Ficus* tree provides a compact syconium (inflorescence) habitat for the lives of a complex assemblage of Chalcidoid insects. These diverse fig wasp species have intimate ecological relationships within the closed world of the fig syconia. Previous surveys of *Wolbachia*, maternally inherited endosymbiotic bacteria that infect vast numbers of arthropod hosts, showed that fig wasps have some of the highest known incidences of *Wolbachia* amongst all insects. We ask whether the evolutionary patterns of *Wolbachia* sequences in this closed syconium community are different from those in the outside world. In the present study, we sampled all 17 fig wasp species living on *Ficus benjamina*, covering 4 families, 6 subfamilies, and 8 genera of wasps. We made a thorough survey of *Wolbachia* infection patterns and studied evolutionary patterns in *wsp* (*Wolbachia* Surface Protein) sequences. We find evidence for high infection incidences, frequent recombination between *Wolbachia* strains, and considerable horizontal transfer, suggesting rapid evolution of *Wolbachia* sequences within the syconium community. Though the fig wasps have relatively limited contact with outside world, *Wolbachia* may be introduced to the syconium community via horizontal transmission by fig wasps species that have winged males and visit the syconia earlier.

## Introduction

*Wolbachia* (Alphaproteobacteria) are maternally inherited endosymbiotic bacteria that are found in arthropods and filarial nematodes [1], [2]. A meta-analysis estimates that the proportion of *Wolbachia*-infected arthropod species is 66% and that there are some 1 million infected species in insects alone [3]. Recently, with a more appropriate data set from both a broad range of species and a sufficient number of individuals per species, this number was reduces to 40%, however confirming that *Wolbachia* are the most abundant endosymbionts among arthropod species [4]. Besides the pandemic distribution, host reproductive manipulations are also important features of *Wolbachia*. The bacteria spread and persist in some host species by manipulating host reproduction via methods including feminization of genetic males [5], parthenogenesis induction [6], killing of male progeny from infected females [7] and cytoplasmic incompatibility [8]. These all provide relative reproductive advantages to infected females, thereby favoring the persistence and spread of the *Wolbachia* strain in host populations [9].

Vertical transmission from mother to offspring via the egg cytoplasm is thought to be the predominant mode of *Wolbachia* transmission within a host species [10]. However, horizontal transfer of *Wolbachia* has also been detected both within and among different host species in many cases. Various findings suggest the existence of horizontal transfer. For example, closely related bacterial strains can be found in taxonomically unrelated hosts [11], [12], [13]; the phylogeny of *Wolbachia* is often strongly incongruent with that of their hosts [12], [13], [14], [15], [16], [17]. Further, it is possible to transfect *Wolbachia* from native hosts to other species, although a newly acquired strain often cannot maintain high infection rates over many generations [18], [19], [20]. A case study in the wasp genus *Nasonia* indicated that the four closely related species were infected with eleven different *Wolbachia* strains, and that horizontal transmission, hybrid introgression and codivergence during host speciation all contributed to the current infection patterns [21]. Though the exact transfer mechanisms are not well understood, many factors may play a part in the process, including close phylogenetic relationship, physical intimate contact or ecological association [17], [19], [22], [23]. Recent work has also shown that host plants may be conduits of bacterial symbionts between insect herbivores that feed upon them [22], [24].

In order to investigate the transmission of *Wolbachia* or its effects on hosts, many studies have compared samples from different localities, in some cases even among different continents [2], [3], [25], [26]. Other studies have focused on ecological communities or at least interacting host species, such as social hosts [27], insects with host-parasite relationships [19], or otherwise linked through feeding on the same food [17], [22]. These species are phylogenetically or ecologically closely related, and even form communities in some cases. However the environment they occupy is open, so they have many opportunities to interact with a wide range of species that is hard to define. This can make it difficult to define the boundaries of a relevant panel of potential hosts from which they may acquire *Wolbachia* infections. However, the diverse but closed communities of fig wasps (Hymenoptera: Chalcidoidea), living within the syconia of fig trees (*Ficus*: Moraceae) are different.

Fig and fig wasps form a classical model in the study of co-evolution and, increasingly, community ecology [28], [29], [30], [31]. We suggest that they may also provide a good model to study infection patterns and transfer routes of *Wolbachia*. Figs are plants in the genus *Ficus*, with around 750 species worldwide, mostly in the tropics. Figs and fig-pollinating wasps are obligate mutualists that have coevolved for over 60 million years [32]. Figs have unique closed inflorescences called syconia, in which the fig wasps (including pollinating and non-pollinating species) reproduce and develop. One fig tree species may host several (and up to 30) different wasp species [33], [34]. Most of the wasp species are chalcidoids from the families Agaonidae, Pteromalidae, Eurytomidae, Torymidae and Orymidae. Different wasp species can survive within the same syconium by employing different living strategies, such as using different flower layers [35], [36], laying eggs at different phases of fig maturity [30], [37] and having diverse larvae diets (e.g. gallers and parasitoids) [30], [38], [39], [40], [41], [42], [43], [44].

The syconia provide fig wasps with a compact habitat isolated from the outside world. The female wasps come out from their natal figs to lay eggs into the ovaries of some flowers in receptive figs. Some of the species (all the pollinators and some non-pollinators) enter the syconia to oviposit, while most of the non-pollinators do not enter syconia but inject eggs through the fig wall. The larvae of the wasps can feed on either fig flowers or parasitize other fig wasps. Upon maturation, the wingless males of most species mate with females inside the syconia, which they do not leave. Meanwhile, other non-pollinator species have fully winged males, which emerge from the syconia and search for females after dispersal.

Previous surveys of fig wasps have proved that the incidence of *Wolbachia* infection in fig-associated wasps is significantly higher than for a broad collection of insects [11], [25], [26]. The compact and closed world of the syconium is one in which intimate physical contact and close ecological association between species are inevitable. Together with the relatively close phylogenetic relationships of these fig wasps species, this suggests that horizontal transmission of *Wolbachia* is more likely to occur in this species community than other less closed systems. The frequent horizontal transmission of *Wolbachia* may also lead to the high evolutionary rates of *Wolbachia* genes, for example the recombination events of outer membrane protein genes, due to the immune responses of the hosts. In the present studies, we are very interested in the following questions:

1. What is the *Wolbachia* infection pattern across all fig wasp species associated with *Ficus benjamina*?
2. Are there more frequent horizontal transfer events of *Wolbachia* among these wasp species living in *Ficus benjamina* than other previously researched species with some kind of intimate relationships?
3. Do *Wolbachia* infections within a closed syconium community have unique evolutionary characters?
4. Compared to open or semi-open communities, is there a decrease in *Wolbachia* exchange between the relatively compact world of within syconia (fig wasps) and the outside (other insects)?

In the present study, we focused on the 17 species of chalcidoid wasps living on *Ficus benjamina*, covering 4 families, 6 subfamilies, and 8 genera. We made a thorough survey of *Wolbachia* infection patterns by using the *Wolbachia* Surface Protein gene (*wsp*) as a marker. We also observed and investigated the biological characteristics on these fig wasps, based on which we tried to reconstruct horizontal transfer routes of *wsp*.

## Materials and Methods

### Ethics Statement

The sampling of living material involved in our experiments included figs (*Ficus benjamina*) and fig wasps (living in *Ficus benjamina*). All necessary permits were obtained for the field sampling. Collection permits were provided by the Institute of Environment and Plant Protection, Chinese Academy of Tropical Agricultural Sciences and Xishuangbanna Tropical Botanical Garden, Chinese Academy of Science.

### Ecological Observations and Fig Wasp Sample Collection and Identification

*Ficus benjamina* occurs naturally throughout Southeast Asia. The oviposition timing of the fig wasp species associated with *F. benjamina* was observed and analyzed based on three individual fig trees. Around 1000 fig syconia were chosen on each tree. The visiting time of all the wasp species was noted in relation to the developmental stage of the fig syconia.

All the fig wasps for molecular experiments were collected from different *Ficus benjamina* trees in Hainan and Yunnan province in the years of 2006–2011. Before the emergence of fig wasps, mature fig fruits were collected and then dissected in the laboratory for fig wasp specimen’s collection. Female fig wasps species were taxonomically determined by morphological diagnostics by using Nikon SMZ80 microscopes, with most of the species described in a previous study in our lab which have corresponding data for both morphological and molecular characters [45]. We tried to maximize the independence of samples by choosing samples from different batches of syconia so as to avoid sampling with an unusual *Wolbachia* distribution. The fig wasp species used are listed in table S1.

### DNA Extraction

Total genomic DNA was isolated from the whole body for each individual sample by using Easypure genomic DNA Extraction kits (TransGen, Beijing, China). The quality of the templates were confirmed by amplification of a partial fragment of COI gene with around 700 bp length (Primers: LCO1490: 5′-GGTCAACAAATCATAAAGATATTGG-3′ and HCO2198: 5′-TAAACTTCAGGGTGACCAAAAAATCA-3′), and the DNA templates with poor quality were discarded. All of the specimen and DNA vouchers were deposited in College of Plant Protection, Shandong Agriculture University in China.

### PCR Amplification and Sequencing

The samples were first screened for *Wolbachia* infection by PCR amplification with the primers wsp81F (5′-TGGTCCAATAAGTGATGAAGAAAC-3′) and wsp691R (5′-AAAAATTAAACGCTACTCCA-3′) that amplified part of the *Wolbachia* Surface Protein gene (*wsp*) [46]. Genomic DNA from *Ceratosolen solmsi* (Hymenoptera: Chalcidoidea), a pollinator of *Ficus hispida*, which had high infection titre of *Wolbachia* was used as positive control, while ddH_2_O was used as template for a blank control. Because in some cases, the amplification of wsp81F and wsp691R could not get a single band, another pair of primers were used, wsp1f (5′-AATAAGTGATGAAGAAACTAG-3′) and wsp691R. The locality of wsp1f is on the right of wsp81F by 3 bp, with 18 nucleotides exactly the same as wsp81F. Both of them do not have degenerate sites. A BLAST search on wsp81F of NCBI GenBank, we found subjects with identical wsp81F sequences also have the same inner 3 bps as wsp1f. So we consider them to have almost the same sensitivity. Touchdown PCR programs were used: 5 min at 94°C, followed by 20 cycles of 30 s at 94°C, 45 s at 56°C (with 0.5°C reduction every cycle), 1 min at 72°C, and then 8 cycles of 30 s at 94°C, 45 s at 46°C, and 1 min at 72°C; and a final elongation step of 10 min at 72°C. PCR products were purified by EasyPure PCR purification Kit (TransGen, Beijing, China) and directly sequenced by ABI3730 capillary autosequencer (Biosune, Shanghai, China). If the sequencing results showed multiple peaks, the PCR products were cloned with Peasy-T1 vector (TransGen, Beijing, China), and 3 positive clones were sequenced. In case the clones showed high sequence diversity, more clones were sequenced. On average, 5–6 clones were sequenced in each multiple infected individual. The length of *wsp* sequences is from 552 to 573 bp with primers of wsp1f and wsp691R and from 555 to 576 bp with primers of wsp81F and wsp691R. In order to acquire accurate infection frequency in each species, negative samples of *wsp* were double-checked by amplification of another two genes, *ftsZ* and *16S rDNA*.

For multilocus sequence typing (MLST), we only selected the samples whose *wsp* sequence was consistent with a single *Wolbachia* infection, and a single clear single gel band was obtained. We used a system of MLST primer F3/R3 nested with MLST standard primers, with the primer sequence and PCR conditions as described on the *Wolbachia* MLST website (http://pubmlst.org/wolbachia/info/protocols.shtml). We also tried to acquire MLST sequences from low *Wolbachia* titre samples, but it was difficult to successfully amplify all the five gene fragments.

### Sequence Analysis

#### Raw sequences treatments, *wsp* typing and ascertainment of recombinants

If multiple identical sequences were obtained for each species, we chose only one to represent them. The sequences were then submitted for *wsp* typing (http://pubmlst.org/wolbachia/wsp/). According to the typing results, the sequences were clustered into different groups of non-recombinant or recombinant *wsp* types. The suggested recombinant evidence of the recombinant *wsp* sequences were further ascertained by using the programs of RDP, GENECONV, MaxChi, BootScan, and SiScan in RDP3 software [47].

#### Phylogenetic analyses

The non-recombinant *wsp* sequences were aligned to relevant sequences previously published on NCBI with Clustal W in Bioedit [48]. Neighbor-Joining trees were constructed by using p-distance model in Mega 5 [49]. Gaps and missing data were pairwise deleted. Bootstrap values were calculated from 1000 replications.

#### Statistic analyses

All of the statistic comparison analyses were based on Chi square test with P values larger than 0.05 indicating insignificant divergences.

## Results

### Ecological Observations on the Fig Wasps Associated with *Ficus benjamina*

*Ficus benjamina* is monoecious (subgenus: *Urostigma,* section: Conosycea). The diameter of syconia is from 12 mm to 25 mm. This tiny space both produces seeds and houses many species of fig wasps. *Ficus benjamina* can represent a common and classical delegate of the fig fruits, so studies of the *Wolbachia* infection patterns in this community can give us a broad impression on the system of fig and fig wasps.

We sampled a total of 17 fig wasp species, which belong to 4 families and 8 genera. *Eupristina koningsbergeri* Grandi is a pollinator, while the others are non-pollinators (table S1) [45]. Wasp number and species composition are very similar among the trees observed. In principle, more wasps are produced in raining season (May to October) than in dry season (November to April). However, the species composition is relatively stable all the year round. Pollinators occupy half of the community population, while the rest are non-pollinators. Many species are easily found whereas some species are rare species, such as *Sycoscapter* sp. 2, *Ormyrus* sp. 1, and *Sycophila* sp. 3. Up to 12 species co-occurred in individual syconia, while up to 14 species were collected on the same batch of a fig tree.

**Figure 1:**
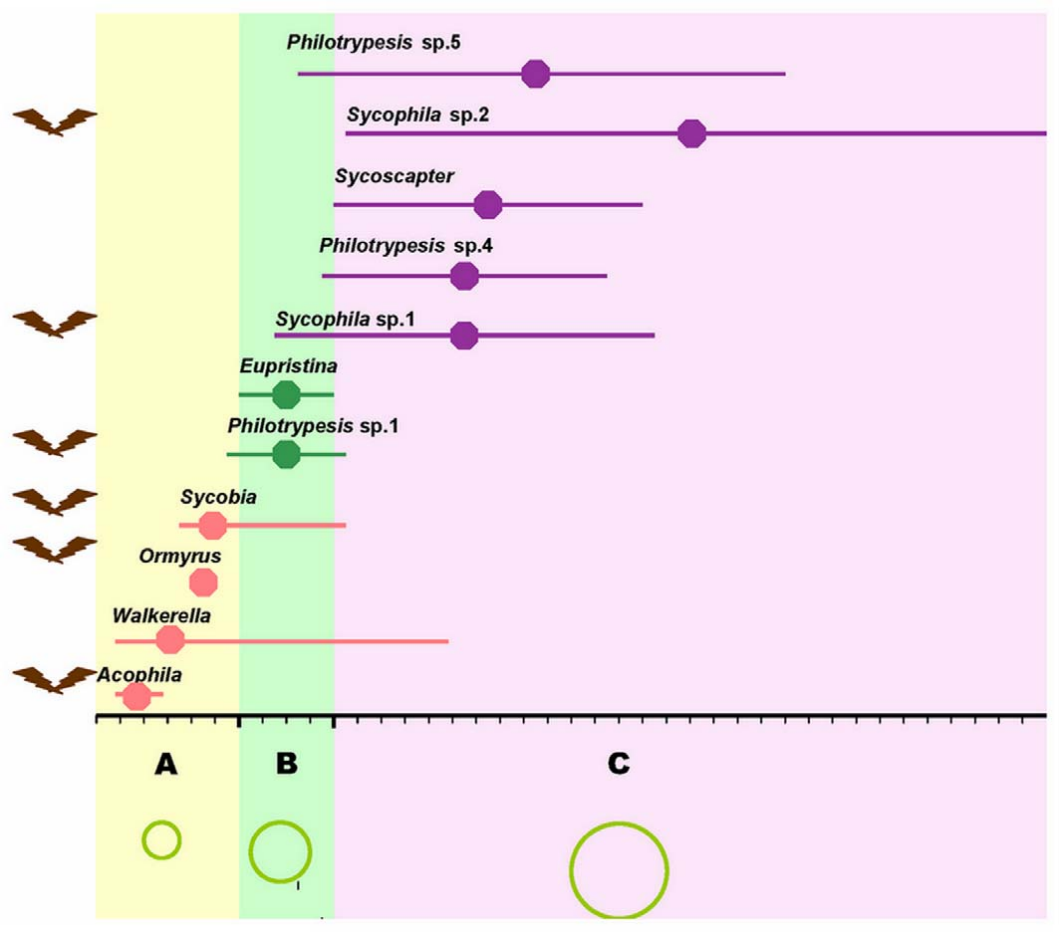
The oviposition timing of fig wasps in *Ficus benjamina*. A, B and C with different ground colors indicate the three different syconia development phases: pre-female phase (Yellow A, ∼6 days), female phase (Green B, ∼4 days), and interfloral phase (Lilac C, ∼30 days). The green circles with different sizes at the bottom indicate the syconia sizes at the different phases. Length of the horizontal line indicates the span of arriving time, and the central point is the hotspot time for the arriving (the arriving individual number is the highest) of each species. Pink, green, and purple lines indicate that the wasps mainly arrive at pre-female, female, and interfloral phase. Brown symbols to the left of the species names mean Species or genus with winged males.

The species assemblage has diverse oviposition behaviors with different species arriving at the figs at different stages of development and using different oviposition sites (Figure 1 and Table S1). Seven species from four genera visit at pre-female stage; 2 species come at female stage, including pollinator *Eupristina koningsbergeri*; 7 species visit at interfloral stage. The detailed order has been described in Figure 1. Except *Eupristina koningsbergeri* lay eggs from inside the syconium, all the others oviposite from outside the syconium. Most of the earlier visitors are prone to be gall makers, while the later are mostly inquilines or parasitoids (Table S1). The species assemblage has diverse larval diets of gall-makers, parasitoids or inquilines, which can indicate the parasitic relationships of some of these species within the same syconium (Table S1).

### High *Wolbachia* Infection Incidence in *Ficus benjamina* Wasps

We screened 870 wasps of the 17 species associated with *F. benjamina* for *Wolbachia* infection. The detailed results are listed in table 1. Fourteen out of 17 species were found to be infected. This infection incidence (84%) is higher than in wider screenings (from many fig species) of fig wasps in Australia (67%) and Panama (59%), but the differences are not significant (*X* ^2^ = 0.031, P>0.05).

**Table 1:** *Wolbachia* infection frequencies of the fig wasp species associated with *Ficus benjamina*.

wasp species	Sample locality	Individuals infected(Individuals screened)	Infectfrequency	Non-recombinant strains	Recombinantstrains
*Eupristina koningsbergeri*	Hainan	64 (96)	0.667	wben-3	rec-3
*Eupristina koningsbergeri*	Yunnan	13 (28)	0.464	wben-2.wHaw.wMors	rec-1.4.16.22.25
*Walkerella benjamina*	Hainan	99 (100)	0.99	wben-2.3	/
*Walkerella benjamina*	Yunnan	13 (18)	0.722	wben-2	/
*Walkerella* sp. 1	Hainan	78 (91)	0.857	wben-2	/
*Walkerella* sp. 1	Yunnan	2 (6)	0.333	wben-2	/
*Sycoscapter* sp. 1	Hainan	95 (120)	0.792	wben-1.3.wHaw.wMors	rec-1.5.12.16.17.21.25
*Sycoscapter* sp. 2	Hainan	6 (6)	1	/	rec-28
*Philotrypesis* sp. 1	Hainan	21 (36)	0.583	wben-1.2.wHaw.wMors.wMel	rec-1.15.16.18.23.25
*Philotrypesis* sp. 1	Yunnan	2 (4)	0.5	wben-2	/
*Philotrypesis* sp. 4	Hainan	10 (59)	0.169	wben-1.2.wHaw.wMors	rec-1.5.6.15.16.19.25.27
*Philotrypesis* sp. 4	Yunnan	26 (37)	0.703	wben-2.wHaw.wMors	rec-4.8.10.14.16.20.21
*Philotrypesis* sp. 5	Hainan	33 (76)	0.434	wben-1.3	/
*Philotrypesis* sp. 5	Yunnan	1 (1)	1	wHaw	/
*Sycobia* sp. 1	Yunnan	34 (38)	0.893	wben-1.2.wHaw.wMors	rec-4.7.9.16.25
*Sycobia* sp. 2	Hainan	10 (31)	0.323	wben-3	rec-1.15.16.24
*Acophila* sp. 1	Hainan	17 (37)	0.349	wHaw	/
*Sycophila* sp. 1	Hainan	0 (11)	0	/	/
*Sycophila* sp. 2	Hainan	28 (36)	0.778	wben-1.2.wHaw.wMors	rec-1.2.15.16.22.25
*Sycophila* sp. 2	Yunnan	1(5)	0.2	/	rec-13
*Sycophila* sp. 3⧫	Hainan	2 (4)	0.5	/	/
*Sycophila* sp. 4	Hainan	0 (22)	0	/	/
*Ormyrus* sp. 1	Hainan	6 (7)	0.857	wben-4	/
*Ormyrus* sp. 2	Hainan	0 (1)	0	/	/

Notes:/indicates no *wsp* sequences were obtained.

⧫in *Sycophila* sp. 3: No *wsp* sequences were obtained, while the infection frequency of 0.50 was identified by the amplification of 16S sequences of *Wolbachia* [1].

1. SimÕEs PM, Mialdea G, Reiss D, Sagot MF, Charlat S (2011) *Wolbachia* detection: an assessment of standard PCR protocols. Molecular Ecology Resources 11: 567–572.

Only three species appeared to be uninfected: *Sycophila* sp. 1, *Sycophila* sp. 4, and *Orymus* sp. 2. However, we screened only one individual for *Orymus* sp. 2. In *Ficus benjamina*, except *Walkerella benjamina* (94.9%) and *Sycoscapter* sp. 2 (100%) with a high infection frequency at nearly 100%, most species had moderate infect frequencies, and the mean infection frequency of different species varied from 16.9% to 89.3%, in contrast to the previously reported “most or few” (>90% or <10%) infection pattern within one species [3]. Infection rates do not differ significantly across all the surveyed species (*X* ^2^ = 1.40E–37, p>0.05).

### *Wsp* genotypes of *Wolbachia* in this Fig Wasp Community

After removing repeated identical sequences within each species, we obtained 103 effective *wsp* sequences from the infected species. These were aligned based on the amino acid motifs of the four hypervariable regions (HVRs) and arranged into four groups: group 1, 45 non-recombinant sequences, 7 genotypes; group 2 & group 3: 57 recombinants, 28 genotypes (the detailed comparison of these two groups were described in the following sections); group 4: one unique sequence (data not shown), 1 genotypes. Evidence of recombination for the 57 recombinant sequences was further confirmed by RDP software (data not shown). All of the sequences were submitted to Genbank with accession numbers JQ342186–JQ342231, JQ361490–JQ361513 and JX192925–JX192928.

### Detection of Seven non-recombinant *Wolbachia wsp* Genotypes

**Figure 2:**
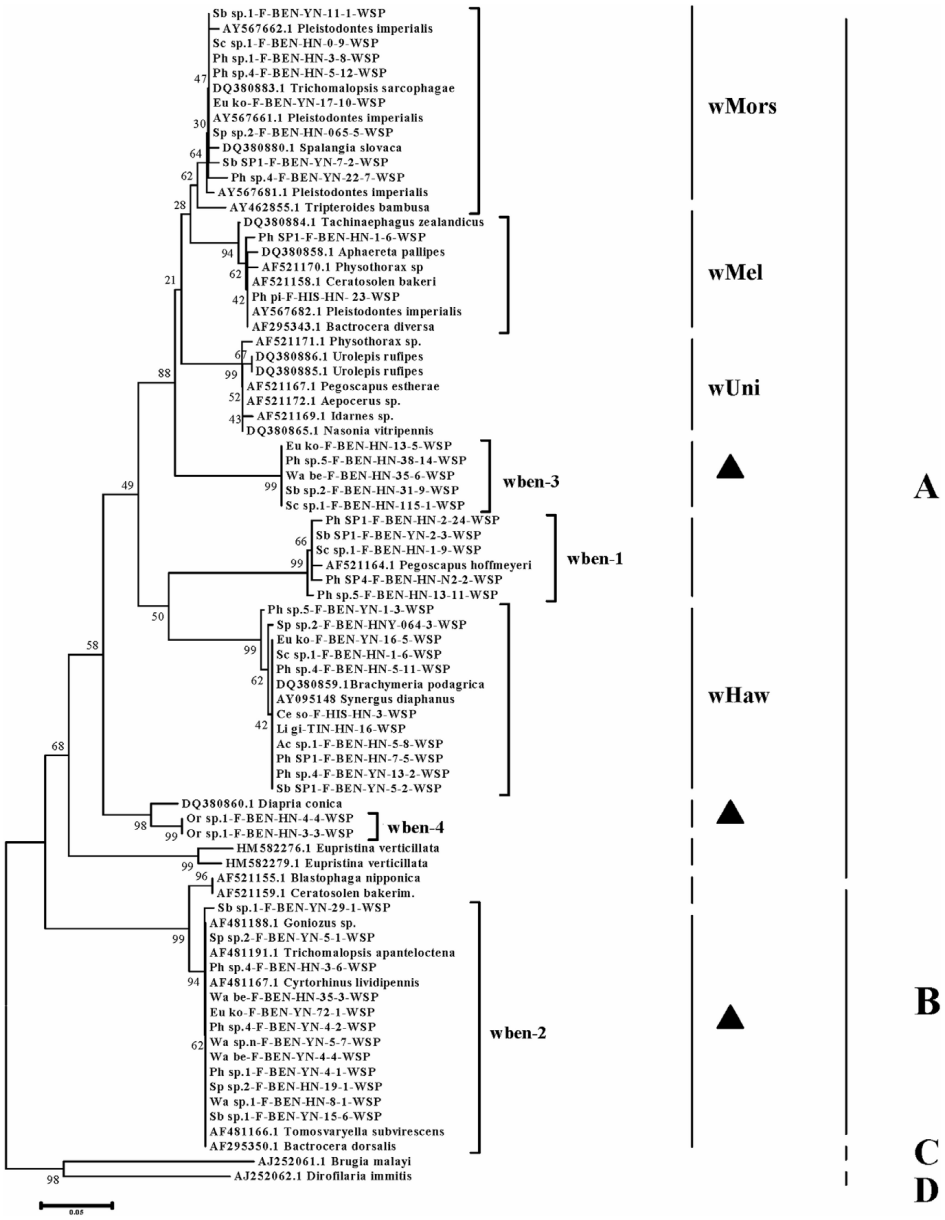
NJ tree constructed with the obtained non-recombinant *wsp* sequences and some related sequences downloaded from GenBank (The accession IDs of the downloaded sequences are annotated on the tree). Two sequences from supergroup C and D are used as outgroups. All of the seven non-recombinant *wsp* types are indicated. The strains to which some *wsp* types belonged with previously reported names are also indicated. The three *wsp* types unique to *Ficus benjamina* are pointed out with ▴. Wben-2 belongs to supergroup B while the other *wsp* types belong to supergroup A.

Based on a Neighbor-Joining analysis of all 45 non-recombinant *wsp* sequences (group 1) with other related sequences, we detected seven non-recombinant *Wolbachia wsp* types based on the sequences similarity, which we named wben-1∼wben-4, wMors, wMel, wHaw (Figure 2), in this fig wasp species community. Within each non-recombinant type, the percentage divergence is from 0 to 0.004, however, between each type, the net percentage divergence is from 0.086 to 0.480. Six belong to *Wolbachia* supergroup A, while wben-2 belongs to supergroup B. Previous studies on *Wolbachia* infection of fig wasps found that *Wolbachia* strain are region-specific. Strains in Asia and Australia are more similar, while they have more divergence from those in the new world, for example, Panama [25], [50]. However in our research, wben-1 is very similar to the *wsp* type infected several fig wasp species in Panama, in contrast to previous results that *Wolbachia* strains infecting fig wasps in Panama and Australia were different and region-specific [25].

Four of the *wsp* types were common ones with wide distributions in other insect hosts besides the fig wasps in *Ficus benjamina*. These were wben-1, wMors, wMel and wHaw, which were found respectively in previous studies [23], [51], [52], [53], [54], [55], [56], [57]. Wben-2, wben-3, and wben-4 were unique *wsp* types first detected here in our *F. benjamina* community. Wben-2 was widely distributed in this community and often co-infects with other types, while wben-3 existed only in some samples from Hainan. Wben-4 was only detected in *Ormyrus* and this host species had no other *wsp* types (Table 1).

We attempted multilocus sequence typing (MLST) of all non-recombinant *wsp* strains that occurred as single infections. This involved use of the five housekeeping genes (*gatB*, *coxA*, *hcpA*, *ftsZ*, and *fbpA*). However, due to the low titre of *Wolbachia*, we only obtained full MLST data for wHaw and wben-2, which can now be identified as ST-19 and ST- 274 respectively (table S2).

### Abundant Recombinant Sequences Exist in *Ficus benjamina*

We detected a large number of recombinant *wsp* types in this study. Of the 57 recombinant sequences, 38 can be assigned to non-recombinant “parents” detected in the *F. benjamina* community (group 2). The origins of the other 19 (group 3) are less clear and involve parent sequences not detected in the fig wasp community. Considering the HVRs composition and origin of all recombinants, we classified them into 28 types, named rec-1∼rec-28 (data not shown). By MLST methods, we only obtained full strain information for rec-28 (Table S2). The distribution of recombinant *wsp* types in the different host species are listed in table 1. All may be functional since they can be translated without frame-shifts or early stop codons.

**Figure 3:**
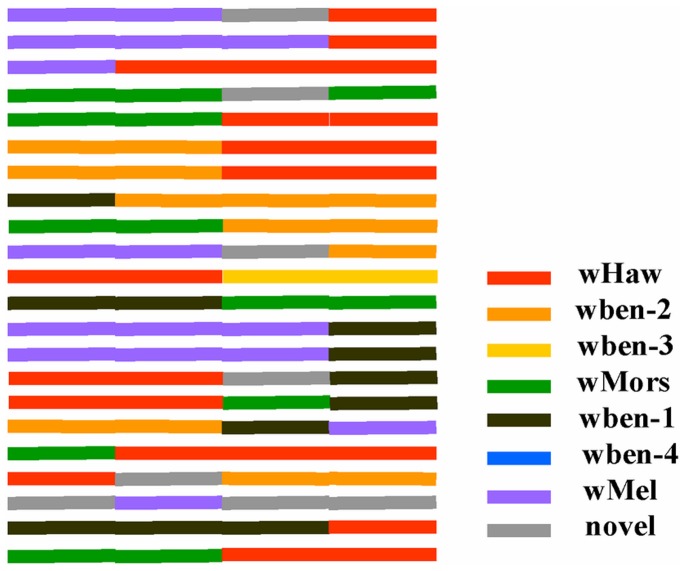
Recombination patterns for the recombinant groups according to HVRs. HVRs of the seven original non-recombinants and some HVR resources not associated with *Ficus benjamina* (we named them ‘novel’.) are indicated with different colors. All the 28 recombinant *wsp* types have different HVRs compositions recombined from the seven non-recombinants or some other HVR resources.

The recombination patterns of the 28 recombinant types according to the origin of the four hypervariable regions are described in Figure 3. Most recombinants (18 of 28) are composed of sequence blocks from two non-recombinant types; 9 recombinants stem from three different non-recombinant genotypes; and rec-24 is composed of sequences from four non-recombinant types. Eight types of the 28 recombinants (rec-1, 4, 10. 15, 20, 21, 26, and 28) are composed of sequences original from not only *Wolbachia* strains of fig wasps on *Ficus benjamina*, but also other host resources we named ‘novel’.

The ubiquitous and *Ficus benjamina*-specific non-recombinant *wsp* types differ in their involvement in recombination. The ubiquitous types with wide range of hosts (wben-1, wMores, wMel and wHaw) are also frequently involved in recombination events. In particular, recombinants involving wHaw sequences were found in all species harboring recombinant sequences. However, the three *F. benjamina* specific non-recombinant *wsp* types are either not involved in any recombination events (wben-4) or involved in few cases (wben-3 and wben-2) (Figure 3).

### Infection Patterns of *Wolbachia* in *Ficus benjamina* Community

Based on a survey of 143 infected wasps, we detected that 63 insects have multiple infections, while 80 were single infected. Superinfected samples were mainly from species *Philotrypesis* sp. 1 in Hainan, *Philotrypesis* sp. 4, *Sycoscapter* sp. 1 in Hainan, *Sycobia* sp. 1 in Yunnan, and *Sycophila* sp. 2 in Hainan. Some species had only single-infected specimens, for example *Ormyrus* sp. 1 in Hainan, and *Sycoscapter* sp. 2 in Hainan. (See table S3).

The species that harbor more recombinant sequences also have more non-recombinants; for example, in *Philotrypesis* sp. 1, *P.* sp. 4, and *Sycophila* sp. 2, implying a positive correlation of recombinants and non-recombinants. Species with multiple *wsp* genotypes mostly harbor both recombinant and non-recombinant types. There is only one exception in *Philotrypesis* sp. 5, which harbors only non-recombinants (wben-3, wben-1). More *wsp* sequences from *Philotrypesis* sp. 5 are required to ascertain this infection appearance. However, there is no indication that the recombinants come from the combination of the non-recombinant sequences hosted in the same individual or species. For example, *Eupristina koningsbergeri* in Hainan hosts non-recombinant wben-3, while it has recombinant rec-3 which derives from wHaw and wMel, not from wben-3 (Figure 3 and table 1).

The infection patterns are independent of sampling localities (*X* ^2^ = 0.19, p>0.05) or the phylogenetic relationships of the hosts (*X* ^2^ = 0.00039, p>0.05). For example, *Eupristina koningsbergeri* in Hainan and Yunnan provinces have different *wsp* types. In contrast, *Philotrypesis* sp. 4 in Hainan and Yunnan has similar infection patterns. Phylogenetically closely related fig wasps may have different infection patterns, e.g. *Sycobia* sp. 1 and *Sycobia* sp. 2. The former has 4 *wsp* types, while the latter has only one, different, *wsp* type. Overall, there are four species in the genus *Sycophila* associated with *F. benjamina*, but only *Sycophila* sp. 2 and *Sycophila* sp. 3 are infected with *Wolbachia* (table 1).

## Discussion

Our study made a thorough survey on the *Wolbachia* infection patterns of all the fig wasp species living inside the closed syconium community of *Ficus benjamina*. We detected high infection incidence in this compact community, as expected. In addition, complicated infection patterns were revealed, including frequent recombination and horizontal transfer, which are signature features of fast evolution of the *wsp* gene [58].

We sequenced 3–6 clones of *wsp* of each multiple infect individual. This method would underestimate the number of multiple infect hosts and the number of strains that co-infect in the individual. Fortunately, large sample numbers will compensate this drawback to some degree. Despite the underestimate, common existing multiple infections in individuals and species is a peculiar character in this system.

### Frequent Recombination Events within the Syconia

WSP in *Wolbachia* is confirmed to be an outer membrane protein [59]. WSP may play a key role in an arms race between arthropod hosts and *Wolbachia*, by inducing host immune responses [60] in which the WSP protein and its surface domains may have a considerable influence. In the dynamic host-bacterium immune interactions, the bacteria can take advantage of the rapidly evolved outer membrane proteins, e.g. WSP, to produce new phenotypes to avoid the host immunity. Recombination may provide an effective means to create new variants and promote the evolution of novel phenotypes, and so far *wsp* sequences have shown the most remarkable pattern of recombination among the *Wolbachia* proteins studied so far [58].

In our *F. benjamina* wasp community, abundant recombinant *wsp* sequences were widely distributed. Recombinants were detected in 53 of the 143 infected fig wasp individuals. At the species level, of the 17 species surveyed, 13 were infected, of which only four lack recombinant infections. Further, all of the recombinant *wsp* sequences were novel and first detected in this focal fig wasp community. Additionally, most of the recombinants were descended from other sequences within the same community, which indicated that most of these recombinants were produced in the syconium community, and further suggested that intimate insect species interactions within the *Ficus* syconia provided a platform for horizontal transfer and recombination of *Wolbachia*.

Several lines of evidence help us to confirm that the recombinants we detected are real recombinants in the cells and not PCR recombinants. First, we have acquired the same recombinants from different host individuals and even from different host species for many *wsp* genotypes. Second, we can confirm recombinants with repeated PCR reactions on the same template. Third, All the recombinants sequences can be translated correctly; if these sequences were PCR errors it is unlikely that all of them have right open reading frame without interruption.

In previous studies, Werren and Bartos reported the first example of recombination and they detected one type of recombinants. The parasitoid wasp and the flies it parasitizes may provide ecological context for this recombination [61]. In Reuter and Keller’s studies, all ants co-infected with four or five distinct *Wolbachia* strains, three of which were arisen by homologous recombination [62]. In *Spalangia* spp. (Hymenoptera: Pteromalidae), 24 out of 48 strains were recombinants [53]. Some statistic analyses predicted that the rate of recombination might be approximately equal to the rate of horizontal transmission [63]. In our studies, 28 types of recombinants occupied 80% (28/28+7) of all genotypes detected within the *Ficus benjamina* community. These comparison results indicated that the recombination events of *wsp* happened frequently in the system of *Ficus benjamina*.

We do not exactly know how recombinants are produced; however, the contact of different *wsp* DNA sequences is a prerequisite. Studies indicate that *wsp* occurs as a single copy in the *Wolbachia* genome, so the co-existence of different *wsp* sequences in a host is evidence of co-infections of divergent *Wolbachia* strains in a host, and the widespread occurrence of co-infections provides a suitable arena for recombination [60].

While the ubiquitous non-recombinant strains were extensively involved in recombination, some non-recombinant strains were involved in no or few recombination events. Why do these strains have different appearances? We suggest that the ubiquitous strains have broad hosts both within the syconium and the outside world, which means that they may be more likely to transfer, so co-infection events are easily formed. However for the unique strains only found within the syconium, they were often present in a host individual with high concentration alone, which may be a function of the host biology so that they do not have chance to recombine.

### Prevalent Horizontal Transfer of *wsp* in *Ficus benjamina* Community

Due to the fast evolution, especially the frequent recombination events, of *wsp*, it is misleading to classify *Wolbachia* strains based only on *wsp* sequences [58], [64]. As *wsp* can be horizontally transferred as a single gene, horizontal transfer of *Wolbachia* strains can not be inferred solely on the basis of *wsp* sequences [58], [64]. However, the horizontal transfer frequency of *wsp* gene itself can indicate the transfer frequency of *Wolbachia* in some degree. Due to the practical difficulty in obtaining the MLST strain information for the *Wolbachia* in these fig wasp species, we only discuss the transfer of the *wsp* gene.

*Wolbachia* spreads across hosts through both vertical and horizontal transfer. Maternal vertical transmission is crucial for the persistence of *Wolbachia* within a single host species from one generation to the next. In our studies, some fig wasp species stably harbor some types of *wsp* sequences, even though the wasps are sampled from different syconia or different fig trees, which indicates that they are not occasional infection events. *Wsp* sequence can exist in the species stably via vertical transmission.

**Figure 4:**
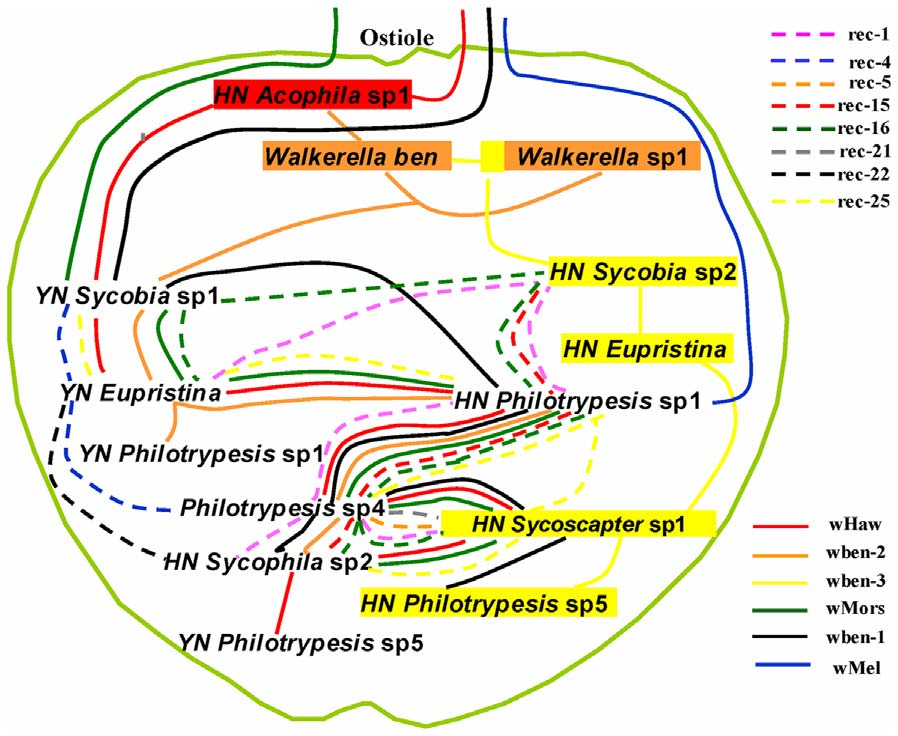
Putative horizontal transfer routes of all the *wsp* types in *Ficus benjamina* community. The outside light-green circle indicates the syconia with the ostiole at the top. The fig wasp species are arranged inside the syconium from the top to the bottom roughly according to their oviposition timing and (or) parasitic relationships. Species with intimate ecological relationships and bear the same *wsp* type are linked by the same line, with dotted and colored lines indicating the recombinant sequences, while full lines representing non-recombinants. Colorful square impressed on species name corresponding to a specific non-recombinant *wsp* type indicates that this species mainly bears the specific *wsp* type. The routes can show that ubiquitous strains 1, 4, 5, and 7 are transmitted from outside world, while the others only exist in this syconium.

Horizontal transfer also appears prevalent in the syconia of *Ficus benjamina*. As shown in Figure 4, we made an assumption on the putative transfer routes of all the *wsp* types infecting more than one fig wasp species. Five of the 7 non-recombinants present horizontal transfer events (except wben-4 which is only present in *Ormyrus* and wMel only in *Philotrypesis* sp.1). Eight *wsp* recombinants are also infecting more than one fig wasp species and representing complicated horizontal transfer patterns within the community.

Though the exact horizontal transfer routes can only be speculated upon, there is plentiful evidence that horizontal transfer is common. First, identical *wsp* sequences exist in species with long distance in phylogenetic relationship. Considering the fast mutation rate of *wsp* sequences [60], it is unlikely that vertically transmitted *Wolbachia* accumulate no mutation in *wsp* sequences among species that diverged a long time ago. Second, there is no clue that the recombinants come from the combination of the non-recombinant sequences hosted in the same individual or species, so horizontal transfer of recombinants among different species seems more likely. It is common that some recombinant sequences exist in different species in the community, but, it is highly unlikely that identical recombinant sequences form independently and repeatedly in different species. Finally, intimate ecological relationships occur among the different fig wasp species such as host-parasitoid, phytophagous insects-food, co-habitant, or close phylogeny relationship and provide good chances for the *wsp* sequences to horizontally transfer within the closed system. Previous studies on dipteran insects associated with mushrooms indicate that the mushroom habitat may provide a somewhat comparable (if more open) ecological arena for the exchange of *Wolbachia* [17]. The wide distribution of ubiquitous *wsp* sequences in the fig wasp assemblage indicates active exchanges of *wsp* between the fig syconia inside world (fig wasps) and the outside (other insects).

We summarized previous studies on the *Wolbachia* infection patterns. These concerned different species, included parasitic insects, social insects, insects within the same community and other phylogenetically closely related insects. The rate of horizontal transfer events of our community was 37.1% (13/37), lower than that in pumpkin community 57% (4/7) and in *Nasonia* 45.4%. If we only consider the non-recombinants in our system, in which 5 out of 7 were involved in horizontal transfer events, the rate was the highest (71.4%).

We also compared the average strain numbers per species harbored in the surveyed insects: the highest one is *Nasonia* (11/4 = 2.75), second parasitoid wasp (48/21 = 2.29), and the third one is our *Ficus benjamina* community (35/27 = 2.06). The above data indicated that parasitic insects were prone to harbor more strains.

Fig wasps in *Ficus benjamina* had 9 species with multiple infections, the rate of which was only lower than the four species of *Nasonia*.

In summary, our species within the syconia of *Ficus benjamina* had abundant events of horizontal transmission, a high number of strains and multi-infections. Compared to those species in the outside world, the close relationship of fig wasps within this closed community may accelerate horizontal transfer events (Detailed iformation was listed in supplementary Table S4).

### Origin of the *Wolbachia* in *Ficus Benjamina*

*Wolbachia* as an endosymbiont presents with a pandemic distribution in arthropods. In our study, we detected that they infected many species of fig wasps. Irrespective of the transmission modes are vertical or horizontal, the origin of the *Wolbachia* in *Ficus benjamina*, especially the non-recombinant strains should be outside of the syconia and they have been introduced inside via certain agents at some time.

Most of the recombinant *wsp*s are descended from the non-recombinants existing in *Ficus benjamina,* so we speculate that these recombinants are original within the syconia, considering the high frequency of recombination events occurring between *wsp* sequences.

How are the non-recombinant *Wolbachia* strains introduced into the fig/fig wasp system? The fig syconium is a relatively closed world isolated from the outside, and fig wasps have limited contact or communications with the outside world. A combined analysis on the ecological results (Figure 1) and the horizontal transferring routes of *Wolbachia* (Figure 4) detect an interesting coincidence: 1) the first stop for the transfer of ubiquitous strains of wHaw, wMors, wben-1 are fig wasp species of *Acophila* or *Sycobia*, which have winged males and visit the syconia very early; 2) ubiquitous strain of wMel is only detected in *Philotrypesis* sp. 1, which also has winged males and arrive at figs very early. So the ubiquitous *Wolbachia* strains may have been introduced inside the syconia via these fig wasp species which may have more opportunities to contact with outside *Wolbachia* sources because of their winged males. The three *Wolbachia* strains unique to *Ficus benjamina* (wben-2, 3, 4) can also be deciphered to have been introduced inside by the fig wasp species with winged males (wben-2, 3 may be introduced by *Sycobia*, while wben-4 only infect *Ormyrus* which also have winged males). However, these unique *Wolbachia* strains can only temporarily be considered as unique to *Ficus benjamina* until they are detected in other hosts. It is also possible that they are indeed *Ficus benjamina* unique strains evolved from an anciently introduced *Wolbachia* strain.

Why are species with winged males more likely the agents helping the transmission of *Wolbachia* from outside into the syconia? In the closed system of fig wasps, most of the males are wingless and they never leave the syconia where they were born. Only very few species have winged males which will emerge from the syconia and mate on the outside of syconium. The mating process is as following: firstly, winged males come out earlier than females. They linger around on the outer surface of the syconia and wait for females. This process can last for about ten minutes (field survey results of our lab). Many other insects such as ants and flies will attack these males and this intimate contact may make them infected by *Wolbachia* from other insects. Then during the mating process, *Wolbachia* can transfer to the reproductive system of the females, which will finally introduce *Wolbachia* into the syconia through their eggs. Though female may spend more time away from syconium, where they will finish the process of mating, fig searching and oviposition, the introduction route of *Wolbachia* is more likely to be: other insects → winged males → females → syconia, rather than: other insects → females → syconia. Because the key factor of successful transmission to the offspring is through the reproductive system of females. The mating behavior outside of the syconia can help the introduction of *Wolbachia* into the syconia through the eggs. Many insects attract females when they are searching for figs or laying eggs, but in these conditions, it may be difficult for *Wolbachia* to infect the eggs in the ovaries and transmit to the next generation.

Current theory favors the idea that females are important factors influencing *Wolbachia* transmission, while males are dead ends of *Wolbachia* vertical transmission. In our studies, only females were included in the PCR screening. However, by combining infection patterns and system ecological data, we suggested that winged males is a proper route to transfer outside strains to the inside world of syconia. Males may also participate in regulating the *Wolbachia* horizontal transmission within the syconia, by the behavior of fighting within the syconium. We suggest that the closed system of *Ficus benjamina* syconia can be used as a model to study the infection of *Wolbachia* in the fig and fig wasp system.

### Conclusions

In conclusion, in the complex fig wasp assemblage sheltered in *Ficus benjamina*, the high infection incidence, frequently occurring recombination events and prevailing horizontal transferring of *wsp*s suggest a rapid evolution of *Wolbachia* in the fig wasp community, and intimate ecological relationship of these fig wasp species within the compact syconia may be the impetus. Though the syconia provide fig wasps with a relatively compact world, they still have active connections (e.g. *Wolbachia* genomes) to the outside world, via few but specific opportunities.

## Supporting Information

Table S1 **Summarization on the observations of some ecological and biological characters on the fig wasps associated with** ***Ficus benjamina.***
(DOC)

Table S2 **Multilocus Sequence Typing (MLST) results on wHaw, wben-2, and rec-28.**
(DOC)

Table S3 **Summarization of superinfection and single-infection specimens.**
(DOC)

Table S4 **Comparison of the** ***Wolbachia*** **infection patterns of fig wasps in** ***Ficus benjamina*** **with previous studies.**
(DOC)
