# Supplementary material for: Chaos of Wolbachia Sequences Inside the Compact Fig Syconia of Ficus benjamina (Ficus: Moraceae)
Source: PLoS One. 2012 Nov 8;7(11):e48882. doi: 10.1371/journal.pone.0048882 (PMC3493598; doi:10.1371/journal.pone.0048882)
Supplement: Table S1 — Summarization on the observations of some ecological and biological characters on the fig wasps associated with Ficus benjamina. (DOC) [file pone.0048882.s001.doc]

Table S1: Summarization on the observations of some ecological and biological characters on the fig wasps associated with *Ficus benjamina*.

| species | Family or subfamily | Oviposition time^#^ | Oviposition site^§^ | Gall type^*^ | Larval ecology |
| --- | --- | --- | --- | --- | --- |
| *Eupristina koningsbergeri* Grandi | Agaonidae  Agaoninae | Phase B | internal | I | galler |
| *Walkerella benjamini* | Pteromalidae  Otitesellinae | Phase A | external | I | galler |
| *Walkerella* sp. 1 | Pteromalidae  Otitesellinae | Phase A | external | II | galler |
| *Sycoscapter* sp.1 | Pteromalidae Sycoryctinae | Phase C | external | I | parasitoids or inquilines |
| *Sycoscapter* sp.2^b^ |  | ∕ | external | I |  |
| *Philotrypesis* sp.1 |  | Phase B | external | I |  |
| *Philotrypesis* sp.4 |  | Phase C | external | I |  |
| *Philotrypesis* sp.5 |  | Phase C | external | I |  |
| *Sycobia* sp.1^a^ | Epichrysomallinae | Phase A | external | III | galler |
| *Sycobia* sp.2 |  | Phase A | external | III | galler |
| *Acophila* sp.1 |  | Phase A | external | IV | galler |
| *Sycophila* sp.1 | Eurytomidae | Phase C | external | III | gall-formers or parasitoids or inquilines |
| *Sycophila* sp.2 |  | Phase C | external | III |  |
| *Sycophila* sp.3^b^ |  | Phase C | external | III |  |
| *Sycophila* sp.4^b^ |  | Phase C | external | III |  |
| *Ormyrus* sp.1 | Ormyridae | Phase A | external | IV | parasitoids or inquilines |
| *Ormyrus* sp.2^b^ |  | Phase A | external | IV |  |

^#^ The oviposition time is determined according to different developing stages of a syconium expanding from receptive phase A to phase C (A: pre-female phase; B: female phase; C: interfloral phase).

^§^ Oviposition site: internal: inside the syconium; external: outside the syconium.

^*^Gall type: I, small and thin; II, small and thick; III, big and thin; IV, big and thick.

^a^ Samples list in BOLD Chinese fig wasps (http://www.boldsystems.org/views/login.php).

^b^ Newly identified species in the present study.

Reference:

1. Wang Z-J, Zhang F-P, Peng Y-Q, Bai L-F, Yang D-R (2010) Comparison of reproductive strategies in two externally ovipositing non-pollinating fig wasps. Symbiosis 51: 181-186.

2. Kerdelhue C, Rossi J-P, Rasplus J-Y (2000) Comparative community ecology studies on old world figs and fig wasps. Ecology 81: 2832-2849.

3. Joseph KJ (1959) The biology of *Philotrypesis caricae* (L.), parasite of *Blastophaga psenes* (L.) (Chalcidoidea: parasitic Hymenoptera). Proceedings XV International Congress of Zoology: 662-664.

4. Tzeng H-Y, Tseng L-J, Ou C-H, Lu K-C, Lu F-Y, et al. (2008) Confirmation of the parasitoid feeding habit in *Sycoscapter*, and their impact on pollinator abundance in *Ficus formosana*. Symbiosis 45: 129-134.

5. Compton SG (1993) An association between Epichrysomallines and Eurytomids (Hymenoptera: Chalcidoidea) in southern African fig wasp communities. African Entomology 1: 123-125.

6. Melika G (2007) Parasitoids (Hym., Chalcidoidea) reared from oak gall wasps (Hym., Cynipidae) in west of Iran, with five new species records. Journal of the Entomological Research Society 9.

7. Gibernau M (2002) Seed predation in *Philodendron solimoesense* (Araceae) by chalcid wasps (Hymenoptera). 163: 1017-1023.

8. Hanson P (1992) The nearctic species of *Ormyrus westwood* (Hymenoptera: Chalcidoidea: Ormyridae). Journal of Natural History 26: 1333-1365.
