# Supplementary material for: Chaos of Wolbachia Sequences Inside the Compact Fig Syconia of Ficus benjamina (Ficus: Moraceae)
Source: PLoS One. 2012 Nov 8;7(11):e48882. doi: 10.1371/journal.pone.0048882 (PMC3493598; doi:10.1371/journal.pone.0048882)
Supplement: Table S2 — Multilocus Sequence Typing (MLST) results on wHaw, wben-2, and rec-28. (DOC) [file pone.0048882.s002.doc]

Table S2: Multilocus Sequence Typing (MLST) results on wHaw, wben-2, and rec-28.

|  | ***gatB*** | ***coxA*** | ***hcpA*** | ***ftsZ*** | ***fbpA*** | **ST** |
| --- | --- | --- | --- | --- | --- | --- |
| **wHaw** | 7 | 6 | 7 | 3 | 8 | 19 |
| **wben-2** | 178 | 157 | 183 | 7 | 89 | 274 |
| **rec-28** | 179 | 155 | 184 | 145 | 17 | 275 |

Notes: Ubiquitous strain wben-1

wben-2 and rec-28 are new ST in MLST database. wHaw is reported in previous research.
