# Supplementary material for: Chaos of Wolbachia Sequences Inside the Compact Fig Syconia of Ficus benjamina (Ficus: Moraceae)
Source: PLoS One. 2012 Nov 8;7(11):e48882. doi: 10.1371/journal.pone.0048882 (PMC3493598; doi:10.1371/journal.pone.0048882)
Supplement: Table S3 — Summarization of superinfection and single-infection specimens. (DOC) [file pone.0048882.s003.doc]

Table S3: Summarization of superinfection and single-infection specimens.

| Fig wasp species | Locality | Specimen  number | NMR | NM | NS |
| --- | --- | --- | --- | --- | --- |
| *Eupristina koningsbergeri* | Hainan | 8 | 1 | 1 | 7 |
|  | Yunnan | 11 | 5 | 0 | 6 |
| *Walkerella benjamina* | Hainan | 2 | 0 | 0 | 2 |
|  | Yunnan | 8 | 0 | 0 | 8 |
| *Walkerella* sp.1 | Hainan | 7 | 0 | 0 | 7 |
|  | Yunnan | 5 | 0 | 0 | 5 |
| *Sycoscapter* sp.1 | Hainan | 8 | 4 | 2 | 4 |
| *Sycoscapter* sp.2 | Hainan | 6 | 0 | 0 | 6 |
| *Philotrypesis* sp.1 | Hainan | 12 | 8 | 3 | 4 |
|  | Yunnan | 1 | 0 | 0 | 1 |
| *Philotrypesis* sp.4 | Hainan | 12 | 11 | 3 | 1 |
|  | Yunnan | 9 | 6 | 3 | 3 |
| *Philotrypesis* sp.5 | Hainan | 6 | 1 | 0 | 5 |
|  | Yunnan | 1 | 0 | 0 | 1 |
| *Sycobias* sp.1 | Yunnan | 12 | 10 | 8 | 2 |
| *Sycobias* sp.2 | Hainan | 6 | 2 | 0 | 4 |
| *Acophila* sp.1 | Hainan | 8 | 0 | 0 | 8 |
| *Sycophila* sp.1 | Hainan | 0 | 0 | 0 | 0 |
| *Sycophila* sp.2 | Hainan | 17 | 15 | 1 | 2 |
| *Sycophila* sp.3 | Hainan | 0 | 0 | 0 | 0 |
| *Sycophila* sp.4 | Hainan | 0 | 0 | 0 | 0 |
| *Ormyrus* sp.1 | Hainan | 4 | 0 | 0 | 4 |
| *Ormyrus* sp.2 | Hainan | 0 | 0 | 0 | 0 |
| Total |  | 143 | 63 | 21 | 80 |

Notes: NMR: Numbers of Multiple-infected specimens (including non-recombinants and Recombinants); NM: Numbers of Multiple-infected specimens (non-recombinants only); NS: Number of Single-infected specimens.
