# Supplementary material for: Chaos of Wolbachia Sequences Inside the Compact Fig Syconia of Ficus benjamina (Ficus: Moraceae)
Source: PLoS One. 2012 Nov 8;7(11):e48882. doi: 10.1371/journal.pone.0048882 (PMC3493598; doi:10.1371/journal.pone.0048882)
Supplement: Table S4 — Comparison of the Wolbachia infection patterns of fig wasps in Ficus benjamina with previous studies. (DOC) [file pone.0048882.s004.doc]

|  | *Taxa* | *Locality* | *Total*  *species number* | *Infect species* | *Multiple*  *infected species* | *Strain*  *number* | *Strain involved*  *in HT* | *Method* | *Reference* |
| --- | --- | --- | --- | --- | --- | --- | --- | --- | --- |
| insect community | fig wasps  in *Ficus. benjamina* | China | 17 | 14 | 9 | 35 | 13 | wsp | Present study |
|  | pumpkin  community | Thailand | 59 | 11 | 0 | 7 | 4 | wsp |  |
|  | rice  community | Thailand | 209 | 49 | 4 | 43 | 3 | wsp |  |
| parasitic insects | *Nasonia* | USA Europe | 4 | 4 | 4 | 11 | 5 | MLST |  |
|  | parasitoid  wasp | Europe  North America | 21 | 15 | 2 | 48 | --- | wsp |  |
| social insect | ants | -- | 23 | 20 | 2 | 6 | 2 | MLST |  |
| other insects | *Agelenopsis*  (spider) | Global | 8 | 8 | 3 | 11 | 2 | MLST |  |
|  | butterflies | India | 56 | 29 | 2 | 15 | 0 | MLST |  |
|  | gall wasps  on oak | Europe | 64 | 11 | 1 | 14 | not clear | wsp |  |

Table S4: Comparison of the *Wolbachia* infection patterns of fig wasps in *Ficus benjamina* with previous studies

Reference:

1. Sintupachee S, Milne J, Poonchaisri S, Baimai V, Kittayapong P (2006) Closely related *Wolbachia* strains within the pumpkin arthropod community and the potential for horizontal transmission via the plant. Microbial Ecology 51: 294-301.

2. Kittayapong P, Jamnongluk W, Thipaksorn A, Milne JR, Sindhusake C (2003) *Wolbachia* infection complexity among insects in the tropical rice-field community. Molecular Ecology 12: 1049-1060.

3. Raychoudhury R, Baldo L, Oliveira DCSG, Werren JH (2009) Modes of acquisition of *Wolbachia*: horizontal transfer, hybrid introgression, and codivergence in the *Nasonia* species complex. Evolution 63: 165-183.

4. Kyei-Poku GK, Giladi M, Coghlin P, Mokady O, Zchori-Fein E, et al. (2006) *Wolbachia* in wasps parasitic on filth flies with emphasis on *Spalangia cameroni*. Entomologia Experimentalis et Applicata 121: 123-135.

5. Frost CL, FernÁNdez-MarÍN H, Smith JE, Hughes WOH (2010) Multiple gains and losses of *Wolbachia* symbionts across a tribe of fungus-growing ants. Molecular Ecology 19: 4077-4085.

6. Baldo L, Ayoub NA, Hayashi CY, Russell JA, Stahlhut JK, et al. (2008) Insight into the routes of *Wolbachia* invasion: high levels of horizontal transfer in the spider genus Agelenopsis revealed by *Wolbachia* strain and mitochondrial DNA diversity. Molecular Ecology 17: 557-569.

7. Salunke BK, Salunkhe RC, Dhotre DP, Walujkar SA, Khandagale AB, et al. (2012) Determination of *Wolbachia* diversity in butterflies from Western Ghats, India, by a multigene approach. Applied and Environmental Microbiology 78: 4458-4467.

8. Rokas A, Atkinson RJ, Nieves-Aldrey J-L, West SA, Stone GN (2002) The incidence and diversity of *Wolbachia* in gallwasps (Hymenoptera; Cynipidae) on oak. Molecular Ecology 11: 1815-1829.
